# Observations upon the Varieties of Deviation, to Which the Last Grinder, or Wisdom Tooth of the Inferior Maxillary Bone, Is Subject, and upon the Accidents Which May Follow Its Growth

**Published:** 1838-05-23

**Authors:** James Trudeau

**Affiliations:** Paris


*Observations upon the varieties of deviation, to which the last Grinder, or Wisdom Tooth of the Inferior Maxillary bone, is subject, and upon the accidents which may follow its growth.* By James Trudeau, M.D.

Dentition of the deciduous teeth is often accompanied by serious disorders, and receives a considerable share of attention from every careful physician. Not so, however, with dentition of the permanent teeth, which commonly takes place without much suffering. I propose, in this paper, to notice briefly the accidents which, sometimes, occur during the growth of the wisdom tooth, and more particularly that of the lower jaw: first, when the space, between the second molar and the base of the coronoid process, is not sufficient to allow of its free growth—secondly, when there is sufficient space, but the tooth takes a wrong direction, first obliquely from behind forwards, and is arrested by the next grinder; secondly, from without inwards along the tongue, causing excoriation of that organ, and impeding its motions; thirdly, from within outwards, so as to penetrate the cheek; fourthly, when the wisdom tooth is detained in the base of the coronoid process; and, fifthly, when it remains covered, at its posterior part, by a hardened gum.

It would be easy for me to multiply these deviations from normal growth, and add many other instances of mal-position of the inferior wisdom tooth, but they may all be easily reduced to the five species just enumerated.

OBSERVATION I.

*The Wisdom Tooth growing from behind forwards, and its growth stopped by the next grinder.*

Madame R——, a young lady, twenty-two years old, felt, three or four months after marriage, a sharp and deep-seated pain in the angle of the inferior maxilla of the left side, the pain soon extending throughout the whole bone. All her teeth were painful, not, however, of the nature of an ordinary toothache. She passed a few months in this state, and as the pain was daily increasing, her physician, suspecting the affection to be rheumatic or neuralgic, resorted to various remedial agents to get rid of it. He began with an antiphlogistic course, by regimen, leeches, poultices, baths, emollient drinks, &c., but without avail. He then used, with no better success, dry and opiate frictions, blisters, and the different antispasmodics. Acupuncturation, a seton at the back of the neck, and the sulphate of quinine internally, were also unsuccessfully tried.

By the advice of the most distinguished physicians of Paris, the lady tried the baths of Bourges, but returned, suffering from excruciating agony. At this time, she consulted Dr. ——, without, however, much hope of relief. When the doctor saw her, her countenance was extremely pallid, she was much emaciated, her strength was impaired, and her appetite gone. For a year, she had been almost literally without sleep, the calm of night bringing with it only the feeling of despair.

Her teeth were sound and white, the gums of a pale rosy colour, and there was not the least appearance of the growth of a wisdom tooth. Examination was, however, directed to this quarter. A deep incision was made in the gum, with a curved bistoury, behind the second large grinder. A small probe being introduced, a hard body was felt. Under the belief, now, that there was a tooth growing obliquely forwards, the growth of which was prevented by the adjoining tooth, the second large grinder was immediately extracted. A few days after this operation, the pain entirely disappeared, and the lady now enjoys excellent health.

M. Esquirol, to whom I communicated this fact, told me that he had cured a young lady, who was insane, of her mania, by the extraction of the second molar tooth, which was, in the same manner, preventing the growth of a wisdom tooth.

To understand affections of this nature, it must be borne in mind, that, when a tooth makes its appearance through the gum, the roots have not attained their entire length. They grow from within outwards, and, if the crown of a growing tooth is stopped by any impediment, during its evolution, the root, increasing in length, by the process of ossification, occupies a place not intended for it by nature, and powerfully compresses the nerves of the part. It is easy, then, to understand the disturbance, caused by a wisdom tooth's being imbedded in the coronoid process, or being arrested by the thickness of the gum, or growing against the next molar, as in this case.

OBSERVATION II.

*The Wisdom Tooth growing from without inwards, on the side of the tongue, and causing an ulceration, of a syphilitic appearance.*

Mr. M——, formerly an officer in the army, living in the country since 1815, came to Paris, with the intention of undergoing an anti-syphilitic treatment, laboring under the idea, that he was affected with the venereal disease. He had had, for some months, an ulceration, at the base of his tongue, on the left side. The movements of this organ were deranged. Mastication was particularly painful. The mercurial treatment, to which he submitted, by the advice of an eminent practitioner of Paris, only aggravated his symptoms. After twenty days' continuance of this treatment, the tongue became enormously swelled, so as entirely to fill the mouth; the gums were in a fungous state, and the breath offensive. This treatment having been suspended, M. M—— applied to Dr. ——. After a very careful examination, he soon discovered, in the maxillary bone, at a distance of about half an inch from the posterior dental canal, a hard body, covered by a floating or moveable portion of the gum. An incision having been made in the gum, a portion of the crown of a wisdom tooth was distinctly visible. The tooth had grown in a wrong direction, and, being in contact with the base of the tongue, had occasioned an ulcer. The tooth was immediately extracted, and a few days after, the ulcer healed up.

OBSERVATION III.

*The Wisdom Tooth growing from within outwards, and penetrating into the substance of the cheek.*

Miss B., twenty-nine years of age, consulted Dr. —— for an immense swelling, on her right cheek, opposite the wisdom tooth, which was exceedingly painful, particularly upon opening the mouth. The doctor suspected at once that this irritation depended on the growth of the last grinder, the crown of which, being directed from within outwards, was penetrating into the substance of the cheek. Upon examination with the finger, this tooth was felt growing in a horizontal direction, and quite imbedded in the muscles. Had it been possible at once to extract the tooth, the patient would have been speedily relieved. But, the swelling of the gums and of the internal part of the cheek, which was ulcerated, interfered with the operation. The tooth was, besides, very much decayed, and would have been broken by any instrument. The indication, then, was to lessen the irritation, which was caused by the crown of the tooth acting as a foreign body. A piece of cork, prepared so as entirely to cover this, and deeply grooved, was placed between the cheek and the teeth. This little apparatus was fastened to the first molar, and remained in its place for three days. Poultices were applied to the cheek, and slightly acidulated gargles used. At the end of the time mentioned, the irritation had subsided, so as to allow the tooth to be extracted, after which, the unpleasant symptoms at once disappeared. These deviations of the wisdom tooth outwards are of common occurrence in practice, but the inclination is generally very slight, and the removal of the tooth not often demanded.

### OBSERVATION IV.

*The Wisdom Tooth arrested in its growth by the base of the coronoid process.*

In this case the right cheek was swelled to an enormous size, the swelling extending from the eyes to the clavicle. The face and neck were covered with numerous abscesses. For twenty months, the man had not been able to open his mouth, and had been fed on liquids, passed through an opening caused by the absence of a tooth. He had, besides, a fistula, at about three inches from the lower angle of the inferior maxilla. A little lower on the neck, there was another. A probe, being introduced into the first fistula, penetrated obliquely, from before backwards, and was stopped by a hard body, supposed to be the root of the third large grinder. From the beginning of the affection, the man's health had been greatly impaired; he was much emaciated, his skin was of a leaden hue, and he suffered much from colics. For a short time past, his digestion had been disordered, and attended with acidity; this, probably, depended on the mixture of his food with the pus, with which his mouth was constantly filled. Various means were resorted to, to open the patient's mouth, and extract the tooth. Leeches, poultices, mercurial frictions, blisters, and compression were used with no better success. Dr. —— now thought of trying a mechanical agent, which succeeded perfectly. It was a conoidal piece of wood, introduced between the dental arch, and pushed in slowly by the patient himself. The following day, the mouth presented an opening of about four lines. A week afterwards, the man's mouth was opened wide enough to allow of the tooth being easily extracted.

A few days after, a necrosed piece of bone was extracted. It proved to be a portion of the base of the coronoid process, on which was moulded or cast a portion of the crown of the tooth. This evidently showed that the tooth had been stopped in its growth by this bone. Since that time the inflammation rapidly disappeared, and in a month the patient was perfectly cured.

### OBSERVATION V.

*The Wisdom Tooth growing under a portion of the gum which cannot be cut by the tooth.*

Orage, a waiter, for a year past had been subject to frequent inflammation of the cheek and fauces,—indeed, ever since the first appearance of the wisdom tooth of the left side. During one of these attacks he was advised to consult Dr. ——. His cheek was swelled, and the tonsil of the left side was inflamed, as well as the fauces. Behind his second large grinder, the crown of a wisdom tooth could be perceived, and covered in its two-thirds by the gum, which was ulcerated. A simple incision through the gum was all that was necessary in this case.

Cases of this description repeatedly occur in practice, and do not always terminate happily. When indurations of the tonsils occur, this gland must be removed.

PARIS, 20th March, 1838.

